# Outcome after severe multiple trauma: a retrospective analysis

**DOI:** 10.1186/1752-2897-7-4

**Published:** 2013-05-15

**Authors:** Christian von Rüden, Alexander Woltmann, Marc Röse, Simone Wurm, Matthias Rüger, Christian Hierholzer, Volker Bühren

**Affiliations:** 1 Department of Trauma Surgery, Murnau Trauma Center, Murnau 82418, Germany; 2 Department of Anesthesiology, University Hospital Würzburg, Wurzburg, Germany

**Keywords:** Severe multiple trauma, ISS, POLO-chart, Post-traumatic stress disorder

## Abstract

**Background:**

Aim of this study was to evaluate prognosis of severely injured patients.

**Methods:**

All severely injured patients with an Injury Severity Score (ISS) ≥ 50 were identified in a 6-year-period between 2000 and 2005 in German Level 1 Trauma Center Murnau. Data was evaluated from German Trauma Registry and Polytrauma Outcome Chart of the German Society for Trauma Surgery and a personal interview to assess working ability and disability and are presented as average.

**Results:**

88 out of 1435 evaluated patients after severe polytrauma demonstrated an ISS ≥ 50 (6.5%), among them 23% women and 77% men. 66 patients (75%) had an ISS of 50-60, 14 (16%) 61-70, and 8 (9%) ≥ 70. In 27% of patients trauma was caused by motor bike accidents. 3.6 body regions were involved. Patients had to be operated 5.3 times and were treated 23 days in the ICU and stayed 73 days in hospital. Mortality rate was 36% and rate of multi-organ failure 28%. 15% of patients demonstrated severe senso-motoric dysfunction as well as residues of severe head injury. 25% recovered well or at least moderately. 29 out of 56 survivors answered the POLO-chart. A personal interview was performed with 13 patients. The state of health was at least moderate in 72% of patients. In 48% interpersonal problems and in 41% severe pain was observed. In 57% of patients problems with working ability regarding duration, as well as quantitative and qualitative performance were observed. Symptoms of post-traumatic stress disorder were found in 41%. The more distal the lesions were located (foot/ankle) the more functional disability affected daily life. In only 15%, working ability was not impaired. 8 out of 13 interviewed patients demonstrated complete work disability.

**Conclusions:**

Even severely injured patients after multiple trauma have a good prognosis. The ISS is an established tool to assess severity and prognosis of trauma, whereas prediction of clinical outcome cannot be deducted from this score.

## Background

According to recent WHO calculations approximately 5.800.000 people die per year as a consequence of trauma [1]. In Germany approximately 80% of traumatic injuries are caused following motor vehicle accidents [2]. In last three decades mortality following multiple trauma decreased from over 40% to 15% as a consequence of improved structural and personal manage and treatment conditions [3,4]. Survival after major trauma may result in life-time psychological distress and/or physical impairment often associated with working disability [5]. Therefore, treatment of severely injured patients with an Injury Severity Score (ISS) ≥ 16 is very challenging both from a medical and also economic point of view [6,7]. The ISS system allocates the Abbreviated Injury Scale (AIS) scores into six body regions and calculates the highest AIS score from the three most severely injured ISS body regions to assign the ISS score in an ordinal scale from 1 = minor to 75 = lethal [8].

Aim of this study was to evaluate prognosis of severely injured patients with an ISS ≥ 50, incidence of severe multiple trauma, trauma mechanisms as well as mortality, invalidity, working ability/disability and quality of life after survival and reconvalescence of major trauma.

## Methods

### Study population

In a retrospective 6-year-cohort-study following the STROBE/EQUATOR checklist patients with severe multiple trauma and ISS ≥ 16 were evaluated in Level 1 Trauma Center Murnau, Germany, between 01/2000 and 12/2005. Due to an internal consensus that usually the cut-off for deadly severe multiple trauma is an ISS of about 40, all Patients with an ISS ≥ 50 (and therefore much higher than the common cut-off) were included in the study. Data analysis focused on pattern of injury, involved body regions, days in intermediate care unit (ICU), clinical outcome, and final outcome of mental condition. Average time of prospective follow up was 3.6 years after trauma (range 18-78 months).

### Data source

Patients´ data was evaluated from data collected by Murnau Trauma Center and transferred to the German Trauma Registry as well as the “Polytrauma Outcome (POLO) Chart” of the German Society for Trauma Surgery, and a personal interview [9]. Written informed consent was obtained from the patients for publication of this report and any accompanying images. Ethical clearance was obtained from Institutional Ethical Committee, and the study adhered to the tenets of the Declaration of Helsinki.

### Abbreviated Injury Scale (AIS)

The AIS classifies injuries in type of anatomic structure, specific structure, body regions, and level and assigns severity in an ordinal scale from 1 = minor to 6 = lethal [8].

### POLO chart

POLO chart includes the *Glasgow Outcome Scale* (1 item), the *Quality of life index* (5 items), the *SF-36* (36 items), and the *Trauma Outcome Profile* (57 items, including the dimensions depression, anxiousness, post-traumatic stress disorder (PTSD), social aspects, pain, body function, activities, and mental function. A multi-dimensional evaluation using the POLO Chart questionnaire was performed in 29 out of 56 survivors with an ISS ≥ 50 to assess health-based quality of life state. All patients who filled out the POLO Chart questionnaire were asked to take part in a personal interview. 13 out of these 29 patients were available by phone and gave their permission.

### Glasgow Outcome Scale (GOS)

The Glasgow Outcome Scale designed in 1975 by Jennett and Bond [10], is an important outcome parameter for further evaluation (including the parameters death, unconsciousness, strong handicap, fair handicap, well recovery), and is evaluated since 2002 as part of the POLO chart. This comparatively simple score became accepted in recent years although criticized due to its numerous subjective variables. 13 out of these 29 survivors with an ISS ≥ 50 additionally underwent a personal interview including four questions about working ability:

1. Are you back in your job again?

2. If yes, do you perform the same job or did you have to change into another job?

3. How long did it take to re-entry into your job?

4. If no, how was level of invalidity (i.e. level of disability of work due to the accident)?

### Statistical analysis

Statistical analysis was performed using SPSS^®^ (SPSS, Chicago, Illinois, U.S.A.), and graphs using Excel^®^ 2010 for Windows XP^®^ (Microsoft, Redmond, Washington, U.S.A.). Results in this study were mentioned as mean values. Significance was statistically calculated based on Pearson's chi-squared test and t-test. A result was considered to be statistically significant with p-value < 0.05. Incidence rates and categorical variables were compared using the Mann-Whitney-test. Reference group included all treated polytraumatized patients with ISS ≥ 16 < 50 in Murnau Trauma Center in the same time period (1435-88 = 1347 patients).

## Results

### Patients and demographic characteristics

In Germany 50.000 people per year suffer a major trauma [11]. Based on this calculation, incidence of severe multiple trauma with an ISS ≥ 50 is about 3.265 patients per year. In our institution 88 out of 1435 evaluated patients after severe multiple trauma (6.5%) demonstrated an ISS ≥ 50. 66 patients (75%) had an ISS of 50-60, 14 (16%) 61-70, and 8 (9%) ≥ 70. 20 patients (23%) were women and 68 (77 %) were men with a age of 40 years (± 17 years, range 18-63) on average. In the group of patients with an ISS ≥ 50, ISS was 56.8 on average, whereas in patients with an ISS < 50, ISS was 24.3 on average. Parameters of all included individuals of both groups (ISS ≥ 50 and ISS < 50) are listed in Table 1.

**Table 1:** Overview of all included parameters of patients after major trauma 2000–2005 comparing both groups, ISS ≥ 50 and ISS < 50

**ISS ≥ 50 vs. ISS < 50**	**ISS < 50**			**ISS ≥ 50**			**Chi-squared test or *t*-test**
**1435 patients**	**number**	**mean**	**%**	**number**	**mean**	**%**	**significance (p)**
Total	1347		93.9%	88		6.1%	
Male	1004		74.5%	68		77.3%	0.83
Female	343		25.5%	20		22.7%	0.66
ISS							
< 20	556		41.3%				
21–40	688		51.1%				
41–49	103		7.6%				
**Mechanism of trauma**							
Car/truck	365		27.1%	31		35.2%	0.23
Motor cycle	212		15.7%	24		27.3%	0.023
Bicycle	96		7.1%	4		4.5%	0.39
Pedestrian	44		3.3%	7		8.0%	0.029
Fall > 3 m	198		14.7%	14		15.9%	0.79
Fall < 3 m	195		14.5%	1		1.1%	0.0012
Other	237		17.6%	7		8.0%	
**Pattern of injury**							
Head/brain	689		51.2%	48		54.5%	0.73
Face	252		18.7%	19		21.6%	0.58
Chest	450		33.4%	49		55.7%	0.0057
Abdomen	211		15.7%	30		34.1%	0.00039
Spinal cord	360		26.7%	30		34.1%	0.27
Pelvis	179		13.3%	27		30.7%	0.00025
Upper limb	311		23.1%	25		28.4%	0.38
Lower limb	315		23.4%	27		30.7%	0.23
Soft tissues	266		19.7%	25		28.4%	0.12
**Primarily unconscious patients (GCS <= 8)**	**245**		**18.2%**	**41**		**46.6%**	**0.0000015**
**Intubation rate**	**648**		**48.1%**	**66**		**75.0%**	**0.0084**
**Reanimation rate**	**18**		**1.3%**	**8**		**9.1%**	**0.0000005**
**Secondarily transferred patients**	**548**		**40.7%**	**36**		**40.9%**	**0.98**
**Preclinical shock (BP systolic < 90 mmHg)**	**73**		**5.4 %**	**16**		**18.2%**	**0,000017**
**Duration until organ failure**		**days**			**days**		
Lung	209	9.5	15.5%	27	9.1	30.7%	0.0028
Coagulation/Blood	44	3.5	3.3%	11	1.7	12.5%	0.000051
Liver	30	11.6	2.2%	2	2	2.3%	0.98
Circulation	178	8.1	13.2%	31	8.1	35.2%	0.0000058
Central nervous system	156	14.9	11.6%	27	20	30.7%	0.000019
Kidney	66	8.9	4.9%	4	5	4.5%	0.89
SIRS	51	8.7	3.8%	11	11.5	12.5%	0.00032
**Duration of intubation**	**944**	**13.4**		**77**	**20.3**		**0.0000094**
**Lethality/Dead**	**179**		**13.3%**	**32**		**36.4%**	**0.0000024**
**Early lethality (< 24 h after hospital admission)**	**46**		**3.4%**	**12**		**13.6%**	**0.000014**
**Time in hospital**		**49.1**			**73.4**		**0.0002158**
**Time on intensive care unit**		**14.4**			**25.9**		**0.0000057**
**Involved body regions**		**2.3**			**3.8**		**3.09444E-29**
**Operations per patient**		**3.6**			**5.7**		**6.25E-08**
**Aim of discharge**							
Home	512		38.0%	23		26.1%	0.12
Rehabilitation hospital	449		33.3%	20		22.7%	0.13
Other hospital	184		13.7%	11		12.5%	0.79
Other	7		0.5%	0		0%	
Dead	179		13.3%	32		36.4%	
Unknown	16		1.2%	2		2.3%	
**Status after discharge due to GOS**							
Well recovered	509		37.8%	20		22.7%	0.036
Moderately affected	92		6.8%	3		3.4%	0.21
Heavily affected	125		9.3%	13		14.8%	0.07
Unconscious	29		2.2%	2		2.3%	0.98
Dead	106		7.9%	20		22.7%	0.00013
Unknown	53		3.9%	4		4.5%	

### Mechanism of injury

**Figure 1:**
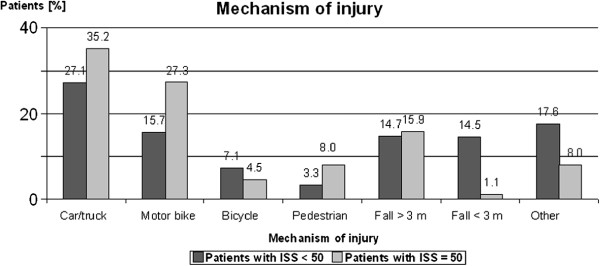
**Mechanism of Injury:** In 27% trauma was caused by motor bike accidents, in 35% by car accidents, in 5% by bicycle accidents and in 16% by fall from a height. In 18%, other mechanisms were causative. Compared with other studies this is a rather high amount. Trauma Center Murnau is located in a holiday region near the Bavarian Alpes, where different kind of sports can be performed including fun sports like paragliding or mountain biking leading to a huge number of different injury mechanisms.

55 out of 88 patients were involved in an accident. In 9 patients suicide was causative and in 24 patients other causes. In 15 patients (27%) major trauma was caused by motor bike accidents, in 19 patients (35%) by car accidents, in 3 patients (5%) by bicycle accidents and in 9 patients (16%) by fall from a height (in 18% of patients no conclusive data were found; Figure 1). Motor cycle accidents were significantly more often causative for multiple trauma with ISS ≥ 50 than ISS < 50 (p-value < 0.05). In all patients trauma was blunt. No penetrating trauma was observed.

### Pre-hospital

18% of patients with ISS ≥ 50 demonstrated pre-hospital shock symptoms, defined as systolic blood pressure < 90 mmHg according to German Trauma Registry, compared with 5% in patients with ISS < 50 (p-value < 0.05).

Primary loss of consciousness at the sight of trauma was observed in 50% of patients with ISS ≥ 50 (Glasgow Coma Scale ≤ 8) compared with 30% in patients with ISS ≤ 50.

Pre-hospital intubation rate was significantly higher in patients with ISS ≥ 50 (75%) compared with patients with ISS < 50 (48%; p-value < 0.05). Cardio-pulmonary resuscitation was required in 9% of all patients with ISS ≥ 50, but only in 1% of patients with ISS < 50 (p-value < 0.05).

Rate of secondary transfer of patients to our national Level 1 Trauma Center was 41% in the group of patients with ISS ≥ 50 compared with 40% in the group of patients with ISS < 50.

### Intensive Care Unit (ICU)

**Figure 2:**
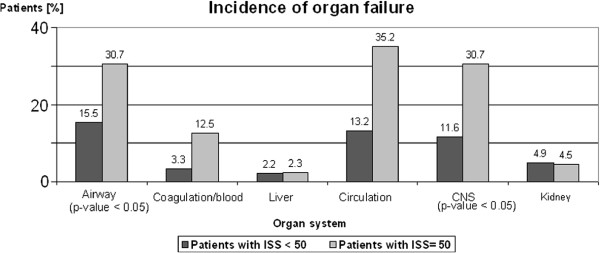
**Incidence of organ failure:** Kind of organ failure is a pivotal parameter for prognosis and outcome in severely multiple-injured patients. Lesions of circulation, airway, coagulation and central nerve system (CNS) play a decisive role in severely multiple-injured patients.

Type of organ failure is a pivotal parameter for prognosis and outcome in severely injured patients. Lesions of circulation, airway, coagulation and central nerve system (CNS) play a decisive role in severely injured patients with ISS ≥ 50 (p-value < 0.05; Figure 2). Rate of organ failure in all patients with ISS ≥ 50 was 54%. In all patients who died following multiple trauma, rate was 78%. Rate of multi-organ failure was 31% in patients with ISS ≥ 50 compared with 10% in patients with ISS < 50 (p-value < 0.05). Sepsis was found in 13% of cases with ISS ≥ 50 compared with 4% in case of ISS < 50 (p-value < 0.05). Duration of intubation/ventilation in ICU was significantly longer in patients with ISS ≥ 50 (20 days on average) compared with patients with ISS < 50 (13 days on average; p-value < 0.05). Mortality of patients with ISS ≥ 50 was significantly higher (36%) than in patients with ISS < 50 (13%; p-value < 0.05). Early mortality in primary cases within the first 24 hours after hospitalization was dependent on ISS. Early mortality in patients with ISS ≥ 50 was significantly higher (13%) than in patients with ISS < 50 (3%; p-value < 0.05).

### Hospital

**Figure 3:**
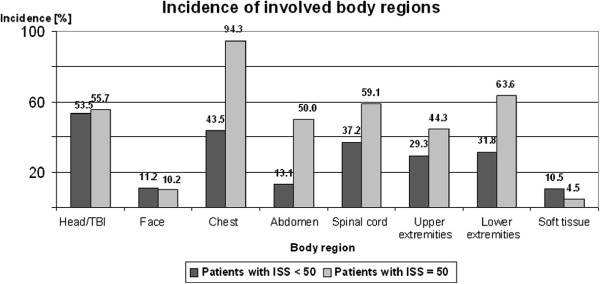
**Incidence of involved body regions:** Injury pattern was very specific in patients with ISS ≥ 50. Mean incidence of involved body regions was significantly higher in patients with ISS ≥ 50 compared with patients with ISS < 50.

**Figure 4:**
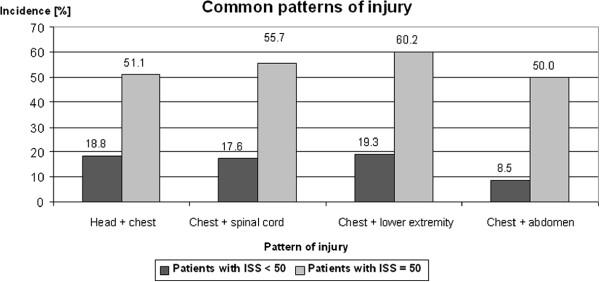
**Common patterns of injury:** In patients with ISS ≥ 50 combined lesions of chest/lower extremities and chest/spinal cord occur significantly more often than in patients with ISS < 50 (mean; p-value < 0.05).

Between 2000 and 2005 duration of hospital treatment of severely injured patients´ time in hospital was 51 days on average including treatment of 15 days in ICU. In patients with ISS ≥ 50 the hospital course was 73 days. These patients were treated 23 days in the ICU and therefore 10 days longer in the ICU compared with patients with ISS < 50 who were treated 49 days in hospital and 13 days in the ICU (p-value < 0.05). Most patients with ISS ≥ 50 were discharged into ambulatory care, 23% to a rehabilitation clinic, 13% were transferred to another hospital and 36% died in hospital. According to ISS regions, in patients with ISS ≥ 50 3.8 body regions were involved compared to 2.3 body regions in patients with ISS < 50 (p-value < 0.05). In 82% of all severely injured patients primary operative intervention was performed in Murnau Trauma Center. On average, 3.6 operations were performed per patient. In all patients with ISS ≥ 50 the severity of injury required primary operative therapy followed by 5.7 consecutive operations (p-value < 0.05). Analysis of the multiple trauma injuries revealed a distinct pattern was very special in patients with ISS ≥ 50: Thoracal lesions were found significantly more often than in patients with ISS < 50 (94% vs. 44%), the same phenomenon was seen in lesions of the lower extremities (64% vs. 32%), the spinal cord (69% vs. 37%), the abdomen (50% vs. 13%), the upper extremities (44% vs. 29%), and the soft tissues (10.5% vs. 4.5%; p-value < 0.05; Figure 3). The most frequent injury patterns in patients with ISS ≥ 50 were combined lesions of chest/ lower extremities (60%) and chest/spinal cord (56%). These combinations of injury occurred more frequently than in patients with ISS < 50 (p-value < 0.05; Figure 4).

### Abbreviated Injury Scale (AIS)

Assessment of severity of organ injury demonstrated an AIS score [12] of 4.2 for lesions of the head, 1.7 for face/neck, 4.1 for thorax, 4.0 for abdomen, 4.4 for spinal cord, 2.1 for upper extremities, 3.9 for lower extremities and 2.0 for soft tissues/other in patients with ISS ≥ 50 compared to an AIS score of 3.8 for lesions of the head, 1.7 for face/neck, 3.3 for thorax, 3.3 for abdomen, 3.4 for spinal cord, 2.1 for upper extremities, 3.0 for lower extremities and 1.6 for soft tissues/other in patients with ISS < 50 (p-value < 0.05).

### Glasgow Outcome Scale (GOS)

38% of patients with ISS < 50 discharged from hospital well recovered, but just 23% patients with ISS ≥ 50 (p-value < 0.05).

### POLO chart, trauma outcome profile, SF-36

**Figure 5:**
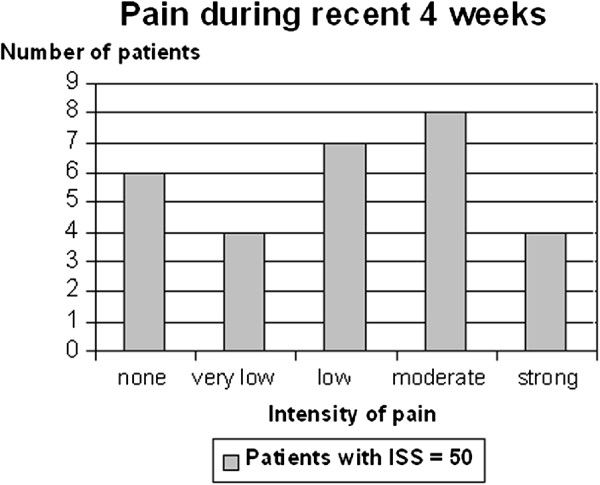
**Pain during recent 4 weeks:** A multi-dimensional evaluation using the POLO Chart questionnaire was performed in 29 out of 56 survivors to get an impression of health-based quality of life state. Time of evaluation was 3.6 years after trauma on average (range 18-78 months). Pain in recent four weeks was evaluated by using a score from “none” to “strong”.

28% of patients reported memory of the sequence of events of the accident. On average 3.6 years after trauma, in 8 out of 29 patients (28%) subjective assessment was bad, and at least moderate in 72%. In 48% impairment of interpersonal contacts following physical and/ or psychological problems and in 62% pain (41% severe pain) was observed (Figure 5). Clinical symptoms of depression (48%), increased anxiousness (45%), posttraumatic stress disorder (41%) and social problems (28%) were observed [13]. In 57% problems in working ability concerning duration, as well as quantitative and qualitative performance were assessed. The more distal the lesions were located (foot/ankle) the more functional disability affected daily life.

### Personal interview

A personal interview was performed with 13 out of 29 patients who had answered the POLO Chart questionnaire. In only 15%, working ability was not impaired. Time to re-entry into work was 24 months on average. 8 out of 13 interviewed patients demonstrated complete work disability.

## Discussion

Outcome after severe multiple trauma is the result of many diagnostic and therapeutic steps over a long time period beginning with the emergency treatment at place of accident until end of rehabilitation. Little is known about final functional outcome of patients after severe multiple trauma. Such information is very important, because severely injured patients often are young, and the majority belongs to the working population [14]. The severity of an injury is one of the parameters determining the outcome of injury. To what extent the severity of an injury affects the outcome is rather unclear yet. It is well known that survival chances of severely injured patients have improved continuously during recent years. Therefore, not only the question of whether, but also how a severely multiple-injured patient survives is of specific interest nowadays ongoing with increasing survival rates. The outcome includes not only physical, but also psychological and social aspects and chronic pain. Studies in the past focused on the influence of psychological or social factors for clinical outcome. Also the pre-traumatic clinical status and accompanying diseases are established factors for prediction of functional outcome in severely injured patients [15]. Our results concerning trauma mechanism showed that an extraordinary amount of severe multiple traumas were caused by motor cycle accidents. Besides, motor cycle accidents were significantly more often causative for major trauma in patients with ISS ≥ 50 than ISS < 50. Our results also showed that in patients with ISS ≥ 50 lesions of the extremities more often were caused by motor bike than by car accidents, which probably is related to a loss of effective protection for these regions of the body. In general, changing of injury patterns during last decades is based on multiple factors: Progress in vehicle and road construction continuously influenced injury pattern and injury severity [16]. While in the Seventies 54% of victims died during hospital stay, in the Nineties 80% of victims died directly at place of accident. Many studies in the past showed that specific mechanisms of injury cause specific injury patterns, which can be diminished by specific security systems. The survivors with ISS ≥ 50 (59 out of 88) were characterized by severe injuries of head/neck, thorax, abdomen and extremities. Their duration of hospitalisation (23 days) was extensively shorter than the results reported by van der Sluis in the Netherlands (30.4 days) and Frutiger in Switzerland [7,17].

Unintentional injuries are responsible for over 3.5 million deaths per year all over the world, making it the sixth leading reason for death [18]. Multi-organ failure/Multi-organ dysfunction syndrome (MOF/MODS) is today´s main causative reason for death of severely injured patients. MOF/MODS can be defined as the development of potentially reversible physiologic derangement involving two or more organ systems not involved in the disorder that resulted in ICU admission, and arising in the wake of a potentially life-threatening physiologic insult [19]. In our study, in severely injured patients MOF was significantly more often seen than in the control group. The trauma registry of the German Society for Trauma Surgery announces the rate of MOF in all polytraumatized patients with 35%, whereas in our hospital an amount of 54% was observed. Therefore, MOF is a decisive parameter for prognosis of clinical outcome. Failure of pulmonary, circulation, blood and/or cerebral nerve system showed an extraordinary amount in severely multiple-injured patients within this study. The blunt traumatic brain injury is known to be the most relevant course of death in this group of patients. Also SIRS was found significantly more often in patients with ISS ≥ 50 than ISS < 50 (12.5% vs. 3.8%). Time of intubation in patients with ISS ≥ 50 was almost twice as long as in patients with ISS < 50. This seems to be explainable by an adequate pain therapy with drug induced breath depression as well as the higher frequency of traumatic chest injuries or the sequels of brain injuries. Additionally, in severely injured patients a high amount of operative revisions were necessary, therefore longer intubation duration avoided patients´ stress related to multiple necessary re-intubations.

In our study, in severely injured patients with ISS ≥ 50 mortality was extensively high (36%), and 15% of these patients were “severely affected” when discharging from hospital. Therefore, with ongoing loss of mortality an increasing number of patients with lifetime handicaps and/or loss of working ability is expectable. Earlier surgical outcome studies focused on body function. From our point of view, for life-time quality after major trauma not only body functions are decicive, but also social, psychological and interpersonal aspects. There are different options to evaluate quality of life by using questionnaires: The global evaluation (e. g. SF-36) and the specific evaluation (e. g. FLQA-d). The POLO-chart questionnaire used in this study is based on a modular data evaluation and therefore a mixture including global and specific parts. Advantages of both methods are combined in the POLO-chart resulting in a maximum of information [20]. Nevertheless, weakening the results of this study is the fact that only severely injured patients with an ISS ≥ 50 were evaluated using this score. In addition, results of scores like POLO-chart generally are just “flashlights” and repetitive evaluations are essential. Also a personal interview is supposed to be a good instrument to get further information about clinical outcome or to answer specific questions of importance. Unfortunately, just 13 out of 56 survivors filled in their phone number in POLO-chart, which is another limiting factor of the results of this study.

Actually, severely multiple-injured patients have a good survival prognosis, but post-traumatic quality of life is still not entirely satisfying. Especially psychological problems and chronic pain lead to a loss of quality in daily life. It is renowned that to know parameters influencing trauma-after-effects is essential for planning, organization, and implementation of rehabilitation programs in a high-specialized facility following major trauma [21]. One knows that about 50% of severely injured patients have one or more chronic problems, and that about 25% do not completely find their way back to work. In our study, a quarter of patients had interpersonal problems, 62% showed chronic pain (41% strong or very strong pain) and 38% of patients are limited in activities of daily life related to pain. Also demographic factors, localization, severity and number of lesions are pivotal determinants of clinical outcome [22,23]. Nevertheless, none of these factors is able to predict which severely injured patients have good chances to recover completely and which not.

In recent literature rate of patients developing PTSD is approximately 10% [24]. In our observation, more than 40% of severely injured patients had beginning symptoms of PTSD. Insofar, PTSD is not just a fatal but rather frequent complication after major trauma, comparable with functional deficits following somatic leasions and/or pain. Our data show that early concomitant psychological therapy is necessary precondition to prevent development of fulminant PTSD. We confirm, that early start of multi-modal therapy concepts including psychological and pain therapy beginning within the ICU is prerequisite to avoid chronic pain syndrome and PTSD.

Concerning body functions more than half of patients declared a loss of function in activities of daily life and working ability. Remarkable was the fact that especially “bagatelle lesions” of the lower extremities were announced to be limiting in activities of daily life. Our data showed that leading leasions and treatment of these “huge” leasions are determining for survival. For functional outcome more often “little” and not perilous lesions result in decicive functional confinements. These findings lead us to begin with an early multi-modal treatment of “bagatelle lesions” including surgical treatment, physical therapy and occupational therapy, because especially these injuries often cause disability in daily life activities. Our results confirm findings of earlier studies that showed a huge amount of functional disability resulting from lesions of the lower extremities [25]. Investing in multi-modal therapy concepts is interesting not even from medical and ethic kind of view, but also under economic aspects in order to reduce the actually high rate of young patients who are unable to find back to work.

In conclusion, we suggest the consequent use of questionnaires like POLO chart or the International Classification of Functioning, Disability and Health (ICF) for early detection of patients with psychological problems, PTSD or problems in social field. These tools are quick, easy to answer and evaluable by commercially available computer programs, and without much effort useful data are obtainable. Thus, the ISS is an established tool to assess severity and prognosis of trauma, whereas prediction of clinical outcome cannot be deducted from this score.

Future studies should compare our local results with the overall German and international trauma registry data.

## Competing interests

The authors declare that there is no actual or potential conflict of interest in relation to this article.

## Authors’ contributions

CVR drafted the manuscript. MRÖ contributed to acquisition of data, analysis and interpretation of data. SW, MRÜ and CH helped to search literature and to draft the manuscript. AW and VB participated in conception, design and coordination, and supervised the whole study. All authors read and approved the final manuscript.

## References

[B1] Mock C Lormand JD Goosen J Joshipura M Peden M Guidelines for essential trauma care 2004 Geneva, Switzerland: WHO

[B2] Guenther S Waydhas C Ose C Nast-Kolb D Quality of multiple trauma care in 33 German and Swiss trauma centers during a 5-year period: regular versus on-call service J Trauma 2003 54 Suppl 5 973 978 12777912 10.1097/01.TA.0000038543.58142.28

[B3] Dresing K Recommended guidelines for diagnostics and therapy in trauma surgery Eur J Trauma 2002 27 137 150

[B4] Barie PS Hydo LJ Fischer E A prospective comparison of two multiple organ dysfunction/failure scoring systems for prediction of mortality in critical surgical illness J Trauma 1994 37 660 666 10.1097/00005373-199410000-00022 7932900

[B5] Pettilä V Pettilä M Sarna S Voutilanen P Takkunen O Comparison of multiple organ dysfunction scores in the prediction of hospital mortality in the critically ill Crit Care Med 2002 30 1705 1711 10.1097/00003246-200208000-00005 12163780

[B6] Neugebauer EAM Tecic T Quality of life after severe injuries Trauma Berufskrankh 2008 10 Suppl 1 99 106

[B7] van der Sluis CK Klasen HJ Eisma WH ten Duis HJ Major trauma in young and old: what is the difference? J Trauma 1996 40 78 10.1097/00005373-199601000-00015 8577004

[B8] Baker SP O´Neill B Haddon W Long WB The injury severity score. A method for describing patients with multiple injuries and evaluating emergency care J Trauma 1974 14 187 197 10.1097/00005373-197403000-00001 4814394

[B9] Pirente N Bouillon B Schäfer B Raum M Helling HJ Berger E Neugebauer E The polytrauma-outcome-(POLO-)chart Unfallchirurg 2002 105 Suppl 5 413 422 12132202 10.1007/s00113-001-0348-5

[B10] Jennet B Bond M Assessment of outcome after severe brain damage Lancet 1975 1 Suppl 7905 480 484 46957 10.1016/s0140-6736(75)92830-5

[B11] Burghofer K Lackner CK Stolpe E Schlechtriemen T Mutschler WE Quality of life five years after severe blunt trauma Notfall Rettungsmedizin 2005 8 552 563 10.1007/s10049-005-0781-z

[B12] Lefering R Trauma score systems for quality assessment Eur J Trauma 2002 2 52 63

[B13] Ettedgui E Bridges M Posttraumatic stress disorder Psychiatr Clin North Am 1985 8 Suppl 1 89 103 3887339

[B14] Meerding WJ Looman CW Essink-Bot ML Distribution and determinants of health and work status in a comprehensive population of injury patients J Trauma 2004 56 150 161 10.1097/01.TA.0000062969.65847.8B 14749582

[B15] Holtslag HR van Beeck EF Lindeman E Leenen LP Determinants of long-term functional consequences after major trauma J Trauma 2007 62 919 927 10.1097/01.ta.0000224124.47646.62 17426549

[B16] Otte D Pohlemann T Wiese B Krettek C Changes in the injury pattern of polytraumatized patients over the last 30 years Unfallchirurg 2003 106 Suppl 6 448 455 14567171 10.1007/s00113-003-0620-y

[B17] Frutiger A Ryf C Bilat C Five years follow-up of severely injured ICU patients J Trauma 1991 31 1216 10.1097/00005373-199109000-00004 1920551

[B18] World Health Organization Global burden of desease 2011 http://www.who.int/healthinfo/global_burden_disease/en/. Accessed on March 1, 2011

[B19] Marshall JC Holzheimer RG, Mannick JA The multiple organ dysfunction syndrome Surgical treatment: evidence-based and problem-oriented 2011 Munich: Zuckschwerdt 21028753

[B20] Pirente N Bouillon B Schäfer B Raum M Helling HJ Berger E Systematic development of a scale for determination of health-related quality of life in multiple trauma patients. The polytrauma outcome (POLO) chart Unfallchirurg 2002 105 Suppl 7 605 10.1007/s00113-001-0348-5 12132202

[B21] Simmel S Bühren V Surviving multiple trauma – what comes next? The rehabilitation of seriously injured patients Unfallchirurg 2009 112 Suppl 11 965 974 19816668 10.1007/s00113-009-1686-y

[B22] Holbrook TL Hoyt DB The impact of major trauma: quality-of-life outcomes are worse in women than in men, independent of mechanism and injury severity J Trauma 2004 56 284 290 10.1097/01.TA.0000109758.75406.F8 14960969

[B23] Brennemann FD Redelmeier DA Boulanger BR Long-term outcomes in blunt trauma: who goes back to work? J Trauma 1997 42 778 781 10.1097/00005373-199705000-00004 9191655

[B24] Breslau N Kessler RC Chilcoat HD Schultz LR Davis GC Andreski P Trauma and posttraumatic stress disorder in the community: the 1996 Detroit area survey of trauma Arch Gen Psychiatry 1998 55 Suppl 7 626 632 9672053 10.1001/archpsyc.55.7.626

[B25] Braithewaite IJ Boot DA Patterson M Robinson A Disability after severe injury: five year follow up of a large cohort Injury 1998 29 55 59 10.1016/S0020-1383(97)00164-2 9659483

